# Multi-Stage 20-m Shuttle Run Fitness Test, Maximal Oxygen Uptake and Velocity at Maximal Oxygen Uptake

**DOI:** 10.2478/hukin-2014-0035

**Published:** 2014-07-08

**Authors:** Giorgos P. Paradisis, Elias Zacharogiannis, Dafni Mandila, Athanasia Smirtiotou, Polyxeni Argeitaki, Carlton B Cooke

**Affiliations:** 1 Department of Athletics, Faculty of Physical Education & Sports Science, National & Kapodistrian University of Athe ns, Greece.; 2 Carnegie Faculty of Sport and Education, Leeds Beckett University, UK.

**Keywords:** aerobic capacity, prediction, testing

## Abstract

The multi-stage 20-m shuttle run fitness test (20mMSFT) is a popular field test which is widely used to measure aerobic fitness by predicting maximum oxygen uptake (VO_2_max) and performance. However, the velocity at which VO_2_max occurs (vVO_2_max) is a better indicator of performance than VO_2_max, and can be used to explain inter-individual differences in performance that VO_2_max cannot. It has been reported as a better predictor for running performance and it can be used to monitor athletes’ training for predicting optimal training intensity. This study investigated the validity and suitability of predicting VO_2_max and vVO_2_max of adult subjects on the basis of the performance of the 20mMST. Forty eight (25 male and 23 female) physical education students performed, in random order, a laboratory based continuous horizontal treadmill test to determine VO_2_max, vVO_2_max and a 20mMST, with an interval of 3 days between each test. The results revealed significant correlations between the number of shuttles in the 20mMSFT and directly determined VO_2_max (r = 0.87, p<0.05) and vVO_2_max (r = 0.93, p<0.05). The equation for prediction of VO_2_max was y = 0.0276x + 27.504, whereas for vVO_2_max it was y = 0.0937x + 6.890. It can be concluded that the 20mMSFT can accurately predict VO_2_max and vVO_2_max and this field test can provide useful information regarding aerobic fitness of adults. The predicted vVO_2_max can be used in monitoring athletes, especially in determining optimal training intensity.

## Introduction

Maximal oxygen uptake (VO_2_max) has been characterized as an important indicator for success in distance running performance as well as for health (Astrand and Saltin, 1967; Hamlin et al., 2012; Noakes et al., 1990). However, the velocity at which VO_2_max occurs (vVO_2_max) is a better indicator of performance than VO_2_max, as it effectively combines both VO_2_max and running economy in one term (di Prampero et al., 1986) and can also be used to explain inter-individual differences in performance that VO_2_max or running economy alone cannot (Billat and Koralsztein, 1996; Billat et al., 2000; Jones and Carter, 2000). Compared to VO_2_max, vVO_2_max is a better predictor for middle and long-distance running performance (Noakes et al., 1990) and it has been reported that it should be used to monitor athletes’ training and to determine optimal training intensity for distance runners (Billat and Koralsztein, 1996; Billat et al., 2000; Laursen and Jenkins, 2002; Smith et al., 1999). Additionally, it seems that vVO_2_max is the minimum speed required to elicit athlete’s VO_2_max, and it is necessary to train at this speed in order to improve VO_2_max and thus aerobic capacity (Billat, 2001; Billat and Koralsztein, 1996; Billat et al., 2000; Hill and Rowell, 1997; Jones and Carter, 2000). Maximizing vVO_2_max through training will increase the running speeds which correspond to a given percentage of VO_2_max and will improve performance since athletes tend to use similar percentages of VO_2_max for a given exercise duration (Jones and Carter, 2000). Thus, vVO_2_max can be very useful for the determination of the intensity of training programs. vVO_2_max is directly measured within a laboratory based test during which running speed increases gradually until the athlete reaches VO_2_max (Billat and Koralsztein, 1996). The direct measurements of VO_2_max and vVO_2_max in a laboratory are time consuming (only one subject at a time) and require relatively expensive equipment, trained personnel and may not be appropriate for some applications. Consequently, there is a demand for quick, inexpensive and valid field tests that can provide a reasonable estimate of VO_2_max. The multi-stage 20-m shuttle run fitness test (20mMSFT) was developed by Léger and Lambert (1982) and it has been used widely since its introduction. The 20mMSFT is a popular field test which is used among athletes of all levels, from children to elite athletes. The 20mMSFT has been used widely for distance runners, soccer players, basketball players, squash, karate and even ice-hockey players in order to measure their aerobic fitness and to predict performance (Paliczka et al., 1987; Ramsbottom et al., 1988; Koklu et al., 2011; Koropanovski et al., 2011; Aslan, 2013). Several studies demonstrated that the assessment of aerobic fitness with the 20mMSFT on a regular basis is beneficial for evaluating the effectiveness of training programs (Castagna et al., 2006; Castagna et al., 2006; Impellizzeri et al., 2005), monitoring soccer players (Castagna et al., 2006), investigating seasonal variations in physiological variables of soccer players (Caldwell and Peters, 2009), basketball players (Ostojic et al., 2006) and ice-hockey players (Geithner et al., 2006). Many studies have reported high correlations (0.90 – 0.93) between performance in the 20mMSFT and VO_2_max (Léger and Lambert, 1982; Paliczka et al., 1987; Ramsbottom et al., 1988; Sproule et al., 1993; Wilkinson et al., 1999). However, as the only outcome of the 20mMSFT is predicted VO_2_max, there is no information regarding vVO_2_max, and consequently no detail on an optimal training intensity for improving performance (Billat and Koralsztein, 1996). The aim of this study was to investigate the validity and suitability of predicting both VO_2_max and the vVO_2_max of adult subjects taking into account performance of the 20mMSFT. As the correlations between VO_2_max and vVO_2_max (Billat and Koralsztein, 1996) and between the 20mMSFT and VO_2_max have been reported as greater than r=0.9, it was hypothesized that the correlation between vVO_2_max and the 20mMSFT should also be of similar magnitude, which should facilitate the product of a predictive equation.

## Material and Methods

Forty eight (25 male and 23 female) PE college students (age = 21.20 ± 1.91 years; body mass = 66.07 ± 11.22 kg; body height = 1.72 ± 0.10 m and % of body fat = 17.40 ± 5.32%) performed, in random order, a laboratory based continuous horizontal treadmill test to determine V0_2_max and a 20mMST, with an interval of 3 days between each test. Testing was performed at the same hour of day ± 2h, with subjects instructed to consume a light meal at least 4 hours before testing and to avoid intense exercise in the preceding 24 hours.

Each subject’s percent body fat was estimated for descriptive purposes using a Harpenden skin-fold caliper (model 68875, UK). Skinfold sites included bicep, tricep, subscupular and suprailiac (Durnin and Womersley, 1974). Prior to participation, subjects received information regarding the design of the study and then gave written informed consent. Prior to the commencement of the research, all procedures involved in this investigation were reviewed and approved by the Athens University’s Research Ethics Committee.

On their initial visit to the laboratory, all subjects were familiarized with the procedures for the treadmill test (Run race 1200, Technogym, Cesena Italy) and for the 20mMSFT. Two days later, participants completed an incremental test to volitional exhaustion with a starting velocity (set during familiarization) of 7–12 km·h^−1^ and 1% slope, for the determination of VO_2_max or VO_2_ peak (dependent on meeting criteria for VO_2_max or not) and vVO_2_max or vVO_2_ peak in an air-conditioned laboratory with the temperature set at 19 – 21° C. Treadmill speed was calibrated previously with a subject running at different speeds while the time it takes for the completion for 30 treadmill revolutions was recorded on a stop watch. Following a 5-min warm-up, the velocity was increased by 1 km·h^−1^ every 3 min from their individual starting velocity until volitional exhaustion. Gas collection was made during the last 60 s period of each 3 min stage in order to allow the subject to attain steady state VO_2_ (Lafontaine et al., 1981). VO_2_ was measured by the open circuit Douglas Bag method. The subject breathed through a low resistance 2-way Hans-Rudolph 2700 B valve (Shawnee, USA). The expired gases passed through a 90 cm length of 340 mm diameter flexible tubing in to 200-liter capacity Douglas Bags. The concentration of CO_2_ and O_2_ in the expired air were measured by the GIR 250 Hitech combined Oxygen and Carbon Dioxide Analyzer (Luton, England). The gas analyzers were calibrated against standardized gases (15.35% O_2_, 5.08% CO_2_ and 100% N_2_). Expired volume was measured by means of a dry gas meter (Harvard) previously calibrated with a 3-liter syringe. Barometric pressure and gas temperature were recorded and respiratory gas exchange data for each work load (i.e. VO_2_, VCO_2_, VE and RER) were determined on a locally developed computer program based on the computations described by McArdle et al. (2010), when VEatps, FECO_2_ and FEO_2_ are known. The highest VO_2_ value obtained during an incremental exercise test was recorded as the subject’s VO_2_ peak which also elicited a heart rate within ±10 bpm of age predicted HRmax and a Respiratory Exchange Ratio (RER) greater than 1.05.

The lowest running speed that elicits a VO_2_ equivalent to VO_2_ peak during the treadmill test was defined as vVO_2_ peak (Billat and Koralsztein, 1996). If the final exercise work load was not completed for 120 s but VO_2_ was increased then vVO_2_ peak was determined from the following equation (Kuipers et al., 1985):

$$\begin{array}{l} {\text{vVO}}_{2}\text{peak}=\text{last}\;\text{work}\;\text{load}\;\text{completd}\;\text{in}\\ 120\;\text{s}+[(\text{time}\;\text{of}\;\text{the}\;\text{uncompleted}\;\text{work}\\ \text{load}/120)*1] \end{array}$$

Fingertip blood samples were taken within 5 minutes of the completion of the test for the determination of lactate levels. To avoid sweat contamination the first drop of blood was wiped off, and only the second was used for analysis. The concentration of lactate was measured enzymatically (Dr Lange, Cuvette Test LKM 140, Hamburg, Germany) using a miniphotometer LP 20 Plus (Dr Lange, Hamburg, Germany). Blood was taken using 10 μl end-to-end capillaries and placed in a reagent solution hemolyzing the blood. Lactate was processed in a reaction producing quinonimin in proportion to the amount of lactate in the sample, and the concentration of quinonimin was read off in an LP 20 Plus apparatus at 540 nm (576 THz) after a 3 min reaction time.

The heart rate (HR) was recorded every 5 s throughout the exercise tests using short-range telemetry (Polar S 710, Polar, Helsinki, Finland).

The 20mMSFT (Handbook, 1983) was administered in a sports hall (temperature 19 – 21° C). It involved running between two lines set 20 m apart at a pace dictated by a recording emitting tones at appropriate intervals. Velocity was 8.5 km·h^−1^ for the first minute, which increased by 0.5 km·h^−1^ every minute thereafter. The test score achieved by the subject was the number of 20 m shuttles completed before the subject either withdrew voluntarily from the test, or failed to be within 3 m of the end lines on two consecutive tones. Heart rates were continuously recorded throughout the test (Polar heart rate monitor S 710), whereas blood lactate was collected 5 min after the test. Scores from the treadmill test and the 20mMSFT were compared using a paired t test. Data were assessed for normality (Kolomonov and Smirnoff) and the relationships between variables for the two tests were calculated using the Pearson’s product moment correlation coefficient. Significance was set at p<0.05.

## Results

**Figure 1:**
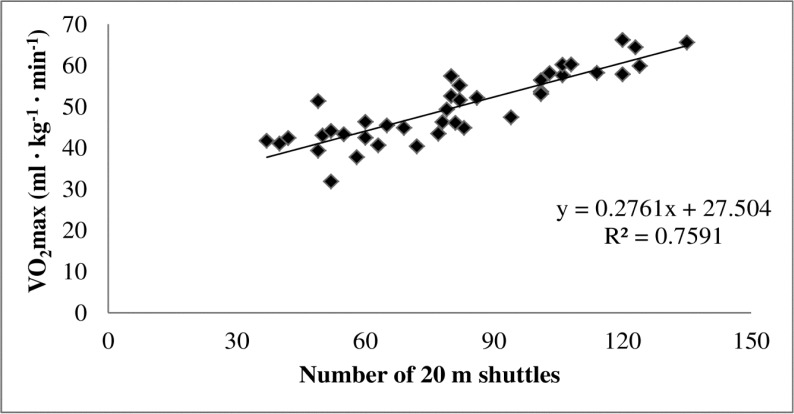
The relationship between the number of multi-stage 20-m shuttle run fitness test and direct measurement of maximal oxygen uptake (n = 48).

**Figure 2:**
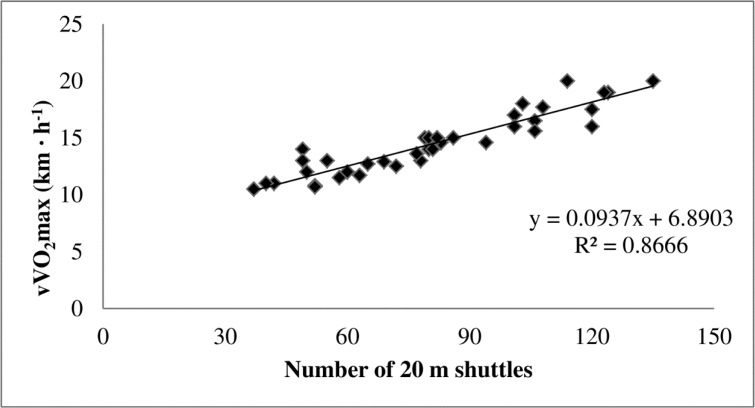
The relationship between the number of multi-stage 20-m shuttle run fitness test and direct measurement of the velocity at maximal oxygen uptake (n = 48)

The analysis of the data revealed statistically significant correlations between the number of shuttles in the 20mMSFT and treadmill VO_2_max (r = 0.87, p<0.05; Figure 1) as well as vVO_2_max (r = 0.93, p<0.05; Figure 2). Additionally, there were no significant differences between the measured and predicted values of VO_2_max (49.98 ± 8.33 and 49.97 ± 7.17 ml·kg^−1^·min^−1^), vVO_2_max (14.52 ± 2.65 and 14.51 ± 2.43 km·h^−1^), HRVO_2_max (194.1 ± 10.03 and 195.2 ± 7.50 beats·min^−1^) and LacuteVO_2_max (12.05 ± 1.96 and 12.09 ± 1.90 mmol·L^−1^). The equation for prediction of VO_2_max was y = 0.0276x + 27.504, whereas for the vVO_2_max it was y = 0.0937x + 6.890.

## Discussion

The purpose of the present study was to investigate the validity and suitability of predicting VO_2_max and vVO_2_max of adult subjects on the basis of performance of the 20mMSFT. The results indicated a high correlation coefficient between shuttles in the 20mMSFT and VO_2_max as well as vVO_2_max. Ideally this type of research should include a two sub-samples design, where one sub-sample is used to obtain the estimation equations and the other sub-sample is used to validate the equations (Morais et al., 2011). However, it can be argued that the present study design is appropriate, as several publications have adopted the same research design (Sproule et al., 1993; Ramsbottom et al., 1988; Paliczka et al., 1987; Stickland et al., 2003; Léger et al., 1988).

The regression model proposed in the present study for the prediction of vVO_2_max is appropriately accurate as indicated the R^2^, the adjusted R^2^ and the standard error of estimation (R^2^ = 0.867, R^2^_adj_ = 0.863 and σ_est_ = 0.980), with only 13% of the variation not explained by this prediction model. While 13% is quite small, some caution should be exercised when this prediction model is used. Similarly, the model proposed in the present study for the prediction of VO_2_max is also appropriately accurate (R^2^ = 0.759, R2_adj_ = 0.753 and σ_est_ = 4.142), even though it is not as strong as the model for vVO_2_max prediction. Again, this prediction model should be used with caution as 24% of the variation is not explained. The unexplained 13% and 24% of variation could be due to factors such as the contribution of anaerobic power and capacity, and/or by some technical error and biological variability.

The correlation between the 20mMSFT shuttles and VO_2_max reported in the present study was similar to values from other studies for adults (Léger and Lambert, 1982; Paliczka et al., 1987; Ramsbottom et al., 1988; Sproule et al., 1993; Wilkinson et al., 1999) but was higher than those for children (Van Mechelen et al., 1986). Statistical analysis also indicated that there were no differences between the direct and indirect values of VO_2_max. These results confirmed the use of the 20mMSFT as a valid predictor of VO_2_max with reasonable accuracy (Paliczka et al., 1987). The results of the present study produced a higher correlation between shuttles on the 20mMSFT and vVO_2_max compared to VO_2_max (r=0.93 and r=0.87, respectively). Additionally, statistical analysis indicated that there were no differences between the direct and indirect values of vVO_2_max. As this is the first study correlating shuttles in the 20mMSFT and vVO_2_max, there are no comparable data available in the literature. However, Paliczka et al. (1987) correlated the 20mMSFT and performance in a 10 km run and reported a similar correlation (r=0.93).

The prediction of vVO_2_max is very useful, especially when it occurs simultaneously for a group of participants through a simple and inexpensive test such as the 20MSFT. Several studies have demonstrated that for well-trained athletes vVO_2_max is the lowest exercise intensity necessary to reach their VO_2_max during a workout, improving their VO_2_max and therefore, aerobic capacity (Billat, 2001; Billat and Koralsztein, 1996; Billat et al., 2000; Hill and Rowell, 1997; Jones and Carter, 2000; Bragada et al., 2010). Moreover, interval training at high intensities, such as vVO_2_max, allows athletes to maintain VO_2_max for a prolonged period of time and leads to a relatively greater improvement in VO_2_max (Demarie et al., 2000). Furthermore, maximizing vVO_2_max through training increases the running speed which corresponds to a given percentage of VO_2_max (Jones and Carter, 2000). This is very important for improving performance since athletes tend to use similar percentages of VO_2_max for a given exercise duration (Jones and Carter, 2000).

The prediction models for the evaluation of vVO_2_max and VO_2_max, aerobic fitness and performance using the 20mMSFT can be used as appropriately accurate tools when direct measurements in a laboratory are not possible due to time and cost restrictions. The findings of the present study provide a relatively quick, inexpensive and valid field test that can provide a reasonable estimate of vVO_2_max, which can be utilized in many different situations. However, some limitations, including the characteristics of the participants, have to be emphasized. The prediction models were derived from testing PE college students with relatively good aerobic fitness. Employment of these models with a different age group (youth or mature athletes), of different capabilities (sedentary, highly trained or world class athletes) and specific sports (e.g. soccer, track & field) has not been tested and the predictive accuracy for these specific situations remains unknown.

It can be concluded that the 20 m multistage shuttle run test can accurately predict VO_2_max and vVO_2_max corresponding to maximal oxygen uptake and this field test can provide useful information regarding the aerobic fitness of adults. Additionally, the predicted vVO_2_max from this test could be used to monitor athletes and determine optimal training intensity in order to improve VO_2_max and thus, aerobic capacity.
